# MAternal Mental Health in the WORKplace (MAMH@WORK): A Protocol for Promoting Perinatal Maternal Mental Health and Wellbeing

**DOI:** 10.3390/ijerph18052558

**Published:** 2021-03-04

**Authors:** Joana Costa, Osvaldo Santos, Ana Virgolino, M. Emília Pereira, Miodraga Stefanovska-Petkovska, Henrique Silva, Paulo Navarro-Costa, Miguel Barbosa, Rui César das Neves, Inês Duarte e Silva, Violeta Alarcão, Ricardo Vargas, Maria João Heitor

**Affiliations:** 1 EnviHeB Lab., Instituto de Saúde Ambiental, Faculdade de Medicina da Universidade de Lisboa, 1649-028 Lisboa, Portugal; jfcosta@medicina.ulisboa.pt (J.C.); avirgolino@medicina.ulisboa.pt (A.V.); mpetkovska@medicina.ulisboa.pt (M.S.-P.); valarcao@medicina.ulisboa.pt (V.A.); 2 Instituto de Saúde Ambiental, Faculdade de Medicina da Universidade de Lisboa, 1649-028 Lisboa, Portugal; navarro-costa@medicina.ulisboa.pt (P.N.-C.); miguel.mgb@gmail.com (M.B.); mjhsantos@netcabo.pt (M.J.H.); 3 Faculdade de Medicina, Universidade de Lisboa (FMUL), 1649-028 Lisboa, Portugal; ines.d.silva@chln.min-saude.pt; 4 Unbreakable Idea Research, Lda., 2550-426 Painho, Portugal; 5 Centro Hospitalar Psiquiátrico de Lisboa, 1749-002 Lisboa, Portugal; maria.emiliacm.pereira@gmail.com; 6 Pharmacol. Sc Depart, Universidade de Lisboa, Faculty of Pharmacy, 1649-003 Lisboa, Portugal; henrique.silva@campus.ul.pt; 7 Instituto Gulbenkian de Ciência, 2780-156 Oeiras, Portugal; 8 CAST—Consultoria e Aplicações em Sistemas e Tecnologia, Lda., 1800-075 Lisboa, Portugal; rcneves@cast.pt; 9 Centro Hospitalar Universitário Lisboa Norte (CHULN), 1649-035 Lisboa, Portugal; 10 Centro de Investigação e Estudos de Sociologia, ISCTE—Instituto Universitário de Lisboa (ISCTE-IUL), 1649-026 Lisboa, Portugal; 11 Consulting House, 1600-477 Lisboa, Portugal; ricardo.vargas@consulting-house.eu; 12 Research Center for Psychological Science (CICPSI), Faculdade de Psicologia, Universidade de Lisboa, 1649-013 Lisboa, Portugal; 13 Departamento de Psiquiatria e Saúde Mental do Hospital Beatriz Ângelo, 2674-514 Loures, Portugal

**Keywords:** biofeedback, cognitive self-control, emotional self-regulation, mental health literacy, perinatal psychological wellbeing, postpartum depression, program evaluation, psychoeducation, work–life balance

## Abstract

Women are exposed to increased burden of mental disorders during the perinatal period: 13–19% experience postpartum depression. Perinatal psychological suffering affects early mother-child relationship, impacting child’s emotional and cognitive development. Return-to-work brings additional vulnerability given the required balance between parenting and job demands. The MAternal Mental Health in the WORKplace (MAMH@WORK) project aims to develop and evaluate the effectiveness of a brief and sustainable intervention, promoting (a) maternal mental health throughout pregnancy and first 12 months after delivery, and (b) quality of mother–child interactions, child emotional self-regulation, and cognitive self-control, while (c) reducing perinatal absenteeism and presenteeism. MAMH@WORK is a three-arm randomized controlled trial. A short-term cognitive-behavioral therapy-based (CBT-based) psychoeducation plus biofeedback intervention will be implemented by psychiatrists and psychologists, following a standardized procedure manual developed after consensus (Delphi method). Participants (*n* = 225, primiparous, singleton pregnant women at 28–30 weeks gestational age, aged 18–40 years, employed) will be randomly allocated to arms: CBT-based psychoeducation intervention (including mindfulness); psychoeducation plus biofeedback intervention; and control. Assessments will take place before and after delivery. Main outcomes (and main tools): mental health literacy (MHLS), psychological wellbeing (HADS, EPDS, KBS, CD-RISC, BRIEF COPE), quality of mother–child interaction, child–mother attachment, child emotional self-regulation and cognitive self-control (including PBQ, Strange Situation Procedure, QDIBRB, SGS-II, CARE-Index), job engagement (UWES), and presenteeism. Intention-to-treat and per-protocol analyses will be conducted; Cohen’s d coefficient, Cramer’s V and odds ratio will be used to assess the effect size of the intervention. MAMH@WORK is expected to contribute to mental health promotion during the perinatal period and beyond. Its results have the potential to inform health policies regarding work–life balance and maternal mental health and wellbeing promotion in the workplace.

## 1. Introduction

Pregnancy and childbirth are transition events that encompass major physiological, physical and psychological changes in a woman’s life. Although it is usually considered a pleasant life stage, the perinatal period (i.e., the period encompassing pregnancy and one year after childbirth; [1]) is often very challenging and requires continuous psychosocial adjustment of the parents-to-be [2], in particular of the mother, who becomes more vulnerable and likely to develop mental disorders [3,4]. Throughout pregnancy, women often experience psychological distress related to rapid changes in body size and shape to which they must adapt, and this can be a potential trigger for body dissatisfaction [5,6]. Such physical changes become intertwined with those at the psychological level. Parents-to-be frequently experience ambivalence during the first trimester of pregnancy, a period during which they adjust themselves to the prospect of parenthood [7]. This effect is more common in women and precedes the mother’s imaginings of her baby and the production of her own representation of the child (the “imaginary baby”). Childbirth poses a challenge to this antenatal representation, requiring that the mother adjusts to the “real baby” [8].

In the first few days after childbirth, approximately 50% of recent mothers are likely to experience postpartum blues [3], which is mainly characterized by transient mild mood disturbance and tearfulness [4,9]. Postpartum blues symptomatology tends to disappear within approximately ten days after childbirth and no medical intervention is usually required [4]. Despite being a common non-pathological condition, postpartum blues has been associated to the onset of postpartum depression (PPD) [3,10,11]. PPD is the most common mental disorder diagnosed among women during the perinatal period [1,12], with an estimated prevalence of 13–19% [13]. PPD symptomatology is indistinguishable from any other non-psychotic major depression episode at a different stage in a woman’s life, the only difference being the onset, as acknowledged in DSM-5 [14]. Depressed parturients experience depressed mood most of the day or loss of interest and pleasure in usual activities, in combination with at least four of the following symptoms: significant weight loss or weight gain, insomnia or hypersomnia, psychomotor agitation or retardation, fatigue or loss of energy, feelings of worthlessness or excessive guilt, diminished ability to think or concentrate, and recurrent thoughts of death or suicide [14]. The onset of PPD is influenced by several factors, such as history of mental disorders [3,4,15], perinatal body dissatisfaction [16], marital satisfaction [17], perceived social support through close relatives and friends [3], life events [15], pregnancy-related complications [15], and hormonal changes [4]. Given the multifactorial nature of PPD and its prevalence and severity, it is crucial to screen for mental disorders during pregnancy and within four weeks of delivery [14].

The short to long-term negative effects of maternal mental disorders on parenting, child physical health, behavior, and cognitive development are well recognized [13,18,19]. Depressed mothers often experience difficulties in establishing affective and protective feelings towards their infant, a process that usually initiates during pregnancy and extends after childbirth [20,21]. Impaired mother–child bonding has been associated with PPD (e.g., [22,23,24,25,26]), with consequences for the emotional and cognitive development of the child that might persist from childhood into adulthood [13]. However, the direction of the association between PPD and impaired bonding should be interpreted cautiously, as poor bonding also occurs among non-depressed mothers [27]. On the other hand, the establishment of a relationship of affection, warmth, trust, and sense of safety from the child towards the mother (i.e., infant–mother attachment) is potentially impaired by PPD. The latter is a relevant predictor of subsequent child externalizing behaviors (e.g., impulsive behavior, hyperactivity, aggression, and delinquency) and poor social outcomes [28].

Available evidence also indicates that maternal mental disorders influence breastfeeding-related outcomes [13], and that mothers suffering from PPD tend to have shorter and earlier ending breastfeeding patterns when compared to non-depressed women [29]. This is of special significance because breastfeeding is one of the earliest postpartum interactions between mother and child, offering a wide range of benefits for both of them [29]. In addition to impaired bonding (characterized by negative affect towards the child and the inability to adequately respond to their demands), mothers with PPD are also less likely to engage in health-related behaviors. Negligent behavior in regard to sleeping practices, as well as increased odds for child abuse have been identified in a literature review addressing the impact of PPD in parenting [13]. All these factors have consequences for the health and wellbeing of the child (e.g., [18,30]).

Children of mothers with persistent depression are at increased risk for behavioral and developmental negative outcomes (e.g., [31,32]). Stein and colleagues have reviewed epidemiological evidence on the effects of disruptive mother–child relationships in children and adolescents in the context of perinatal mental disorders [33]. They found long-term effects of the exposure to perinatal maternal mental disorders on the behavioral competence and cognitive development of these children. For example, exposure to PPD has been described as a relevant predictor of externalizing behaviors, such as inattention and hyperactivity during childhood (e.g., [34,35,36]). As for cognitive development, data from the Avon Longitudinal Study of Parents and Children, United Kingdom, reveals that adolescents of postpartum depressed mothers were 1.5 times more likely to fail mathematics at age 16; ca. 16% of this association was explained by attentional control [37]. Similar results were obtained for receptive vocabulary and inattention for children aged 4 to 5 years that were enrolled in the Canadian National Longitudinal Survey of Children and Youth [38]. Children from postpartum depressed mothers were 1.94 times more likely to display low receptive vocabulary and increased inattention. It should be noted that the association between perinatal maternal mental disorders and offspring negative outcomes is mediated by several factors [33]. For example, after controlling for family background covariates, the effect reported by Letourneau and colleagues on receptive vocabulary was no longer significant [38].

### 1.1. Return to Work after Childbirth

Maternal mental disorders impair the life of a woman as a whole, including her functioning in the workplace [13,39]. Healthy and motivated employees are fundamental at the organizational level and for the socioeconomic wellbeing of any country [40]. Engaged employees are less likely to experience absenteeism or presenteeism, thus contributing to increased productivity [41]. In addition to individual (e.g., health conditions, and resiliency) and family (e.g., spouse support, and financial strain) characteristics, the workplace in all its dimensions (i.e., interpersonal relations and group dynamics, organizational culture, productivity and quality/safety-related challenges, and external environment) represents a potentially major stress factor to employees [42].

During the postpartum period, many women return to paid work. Maternity leave in Portugal is of 42 mandatory days after childbirth. Portuguese parents are also eligible for 120 or 150 days of paid leave at 100% and 80%, respectively. The specificities of maternal and parental leave vary across the European Union-28. For example, German mothers are eligible for up to 42 non-mandatory days during the antenatal period and paid postpartum leaves of 56 mandatory days. In Sweden, parental leave is up to 240 days of paid plus unpaid leave until 18 months postpartum [43]. These differences in national legislation imply that, depending on the country, European women return to work at different stages of physical and psychological postpartum recovery.

Despite latent psychosocial benefits from being employed [44] and empirical evidence that women respond positively to returning to work after childbirth, recent mothers may perceive this moment as a stressful event [45]. In a qualitative study addressing the perceptions of recent mothers when returning to work within one year after childbirth, several resilience-related challenges were identified, the most common one being role conflict (i.e., the difficulty in balancing work and childcare responsibilities) [45]. This arises from three major forms of conflict between roles: time, strain- and behavior-based conflict [46]. For example, a full-time non-flexible work schedule reduces mother’s time with her child, whereas low levels of social support by either her co-workers or partner can result in conflict between work and childcare responsibilities.

Having a child can interfere with career prosecution, and this can represent a discouraging factor to motherhood. Indeed, women in developed countries are having their first child at an older age. In 2018, Portuguese women were giving birth to their first child at age 30.4 vs. 25.0 back in 1960 [47]. Under this scenario, organizational support is fundamental, and the path to a healthy workplace does require the commitment of organizational leaders [48,49]. The continuous motivation and engagement of all employees implies that leaders adopt an empathic mindset towards the different life and career phases of employees. Towards this end, the European Commission has recently approved a new directive on work–life balance for parents and carers—Directive EU 2019/1158 [50]. With regards to parenting, Directive EU 2019/1158 encourages a gender-balanced use of family-related leaves and flexible working arrangements, thus increasing women’s participation in the labour market (by reducing role conflict).

Maternal mental disorders during the perinatal period and beyond represent an issue of clear societal significance. This strongly argues for interventions targeting the prevention rather than the treatment of such conditions. Available evidence indicates that recent mothers who go through psychological and psychoeducational interventions are at a lower risk of developing PPD when compared to women who receive care-as-usual from their health units [39,51,52,53]. As previously mentioned, multiple factors are involved in the onset of PPD, which implies that identifying the most effective intervention type is difficult and context-dependent [51]. Thus, it is not surprising that both individually-based interventions, including home-visiting interventions, and group interventions have proven effective in reducing PPD, either applied during pregnancy or postpartum [39,53]. In addition, interventions making use of wearable devices providing biofeedback have also been implemented. In these cases, physiological signals known to vary under stressful situations [54] are monitored through sensors. The processing of these signals provides information regarding the individual’s physiological activity, which in turn allows her/him to respond accordingly [55]. For example, biofeedback on heart rate variability has been proven effective in reducing depression symptomatology during the perinatal period, as measured by Edinburgh Postnatal Depression Scale scores [56,57].

More recently, mindfulness-based therapy (MBT) interventions have also been implemented to reduce perinatal maternal depression, anxiety and perceived stress [58,59]. Mindfulness refers to the non-judgmental experiencing of everyday life [60], and available evidence shows that MBT may reduce anxiety and depression in clinical populations [61,62]. The few studies that assessed the effectiveness of MBT on perinatal mental health outcomes suggest a reduction in anxiety [58,59] and depression symptoms [63]. To the best of our knowledge, no study combining psychoeducational interventions, including short training sessions of mindfulness as a stress coping mechanism, with biofeedback-coupled monitoring of physiological signals have been implemented in pregnant and postpartum women. This is a promising approach, since it combines cognitive, behavioral, emotional, and physiological dimensions, and these are all known to be involved in depression and anxiety phenomena.

The MAternal Mental Health in the WORKplace (MAMH@WORK) project assumes an integrated approach to the promotion of perinatal maternal mental health and is based on three key aspects. First, maternal mental disorders do not only affect mothers—it affects the physical and mental health and wellbeing of her partner and closest family [13,18,19]. Second, possible negative effects for the child (i.e., externalizing behaviors and cognitive development) may go beyond the perinatal period and extend throughout adolescence and into adulthood [33]. Third, work–life balance strategies are essential to reduce the distress associated with the return to work [45] and to promote wellbeing [64]. To tackle these issues, the MAMH@WORK project proposes to develop, implement and assess the effectiveness of an intervention directed at perinatal mental health promotion. This project will combine e-mental health tools and face-to-face intervention methods to promote maternal mental health starting from late pregnancy until one year after childbirth, including the return to work.

### 1.2. Aims and Objectives

The main aims of MAMH@WORK are: (a) to promote perinatal mental health, from the last trimester of pregnancy up to the first 12 months after childbirth, (b) to foster the healthy psychosocial and emotional development of children via an improved quality of early mother–child interactions, and (c) to reduce sickness absence and presenteeism of working-age women during this period. Considering these aims, the main goals of this project are to develop a maternal mental health and wellbeing promotion program and to evaluate its effectiveness. To achieve these goals, it is proposed the design, implementation and effectiveness assessment of a brief group cognitive-behavioral therapy-based (CBT-based) psychoeducation, including mindfulness e-training, combined with biofeedback. Specific objectives are to (a) design an intervention program to promote wellbeing and prevent perinatal mental disorders in Portuguese pregnant women; (b) develop a smartphone app that will provide women with targeted biofeedback based on the continuous (wearable-based) biomonitoring of selected physiological signals; and (c) assess the impact of the intervention on maternal perinatal mental health outcomes, on the interaction between mother and child (namely regarding the quality of mother–child interaction, child–mother attachment and child emotional self-regulation and cognitive self-control), and on the reduction of women’s absenteeism and presenteeism after childbirth.

## 2. Materials and Methods

### 2.1. Study Design and Setting

This is an open-label, three-arm randomized controlled trial of pregnant women who will be followed throughout pregnancy (starting at 28–30 weeks of pregnancy) until 12 months postpartum. The MAMH@WORK project will be conducted at the Environmental Health Institute of Lisbon School of Medicine (ISAMB-FMUL), Portugal, with clinical data being collected at three primary health care units, three hospitals and in a network of private clinics (all part of the MAMH@WORK consortium).

### 2.2. Sampling Details and Characteristics of the Sample

The target population of this mental health promotion project are pregnant women living in Portugal, with no diagnosis of depression, and employed at the time of recruitment. Women will be recruited at 28–30 weeks of gestational age, since antenatal depression is a relevant predictor for PPD [65,66]; thus, the potential effectiveness of the intervention should increase if it starts during pregnancy. All women booked for delivery at Portuguese hospitals attend various antenatal routine appointments in primary and secondary health care units, which are intended to reduce complications during pregnancy and to improve the chances of positive pregnancy outcomes. These appointments also serve for counselling about health behaviors during the antenatal and postpartum periods. Recruitment will take place in the Lisbon Metropolitan Area from three primary health care units and a network of private clinics. Women at 28–30 weeks of gestation attending an antenatal routine appointment will be invited, by their doctors or nurses, to participate with their babies in the study according to inclusion and exclusion criteria detailed below. In case of agreement, women will be asked to sign a data agreement form and will later receive a formal invitation addressed by the research team. To assess their eligibility to be enrolled in the study, women will be presented with a screening questionnaire conducted by trained psychiatrists and psychologists. In addition, clinical inclusion and exclusion criteria, namely serious mental disorders diagnosed, will be evaluated by antenatal healthcare professionals (i.e., general practitioners, obstetricians, psychiatrists, and psychologists).

To be enrolled in the study, women must meet the following inclusion criteria: native Portuguese speakers or women living in Portugal for at least 5 years; intended pregnancy; primiparous, singleton pregnant women at gestational age of 28–30 weeks; aged 18–40 years; employed; eligible for maternal leave; with access to a smartphone; and have had understood and signed the project’s informed consent form.

Exclusion criteria at the baseline are: diagnosis of (a) a serious mental disorder (e.g., schizophrenia, schizoaffective disorder, other psychotic disorders, bipolar and related disorders, and personality disorders), (b) a chronic disease resulting in functional impairment, or (c) a neurodevelopmental disorder (e.g., autism spectrum disorders and intellectual disability); history of major depression; being under antidepressant medication; alcohol and/or drug addiction; high-risk pregnancy; and fetal malformations. These criteria were defined to ensure that relevant stress-related heterogeneity at the baseline will be reduced in the study. In addition to these exclusion criteria, mother–child dyads will be excluded from the study (after enrolment) in case of preterm birth (less than 37 weeks of gestational age), very-low weight at birth (less than 1500 g), or unexpected newborn malformations.

Randomization will follow the minimization procedure [67] aiming for a balanced sample size for educational level and age groups at the baseline (18–24 years old, 25–34 years old, and 35–40 years old). Random numbers and their assignment will be sealed in envelopes, kept in sequence and opened consecutively by trained researchers as pregnant women are screened for eligibility and enrolled in the study.

The minimum sample size for MAMH@WORK project will be 159, that is 53 women in each group. Attrition rate is considered to be approximately 40% and thus, a total of 225 pregnant women, 75 in each arm, will be enrolled in the study. These calculations are based on a literature review on psychological interventions for PPD prevention, which found mean depression scores (± standard deviation) of intervention and control groups of 11.00 ± 14.63 and 11.52 ± 14.93, respectively, at the final assessment of the study [39]. These sample size calculations assume the average difference between intervention and control arms reported by Dennis and Dowswell [39], considering a 95% confidence interval and a 80% power of the test to detect a difference between depression scores of both arms. This sample size allows for a medium effect size of the intervention to be detected for depression and anxiety scores.

### 2.3. Description of the Intervention

The intervention will be developed in three phases: (1) a scoping literature review followed by a Delphi panel for consensus building on the contents and structure of a brief group CBT-based psychoeducation intervention, (2) the implementation and (3) effectiveness assessment of the intervention. In order to produce culture-specific recommendations for the intervention, Portuguese general practitioners, obstetricians, pediatricians, neurologists, psychiatrists, health and clinical psychologists, and nurses specialized in perinatal mental health care will be invited to take part in the Delphi panel, which will be informed by the scoping literature review.

The intervention will address maternal mental health and wellbeing protection and promotion during late pregnancy, postpartum and return to work. More specifically, it will target women’s psychosocial adjustment and resilience during this life stage, while promoting several strategic skills: recognition of signals and symptoms of mental disorders; reduction of stigma against mental disorders; coping skills for stressful situations, with a special focus on e-training of mindfulness; enhancement of help-seeking efficacy; and strengthening of emotional and cognitive self-regulation. It will follow a cognitive-behavioral and psychoeducational paradigm, and it is planned as a short-term group intervention of 20 h to be delivered in two blocks, one immediately before delivery and the other before returning to work. The first block will consist of two sessions, four hours each, whereas the second block will be organized into three sessions, four hours each. To better meet the objectives of this intervention, group size will not be larger than 15 women per session. Intervention block 1 will also include mindfulness exercises for coping with stress, and short videos with easy-to-follow exercises will be available at the private area of the website (details provided below). Block 2 will recap the main contents of block 1 and focus on resilience-promoting skills for the return to work. Women enrolled in the study will have access to e-mental health literacy materials (details provided below).

A standardized operation procedure (SOP) manual based on the literature reviews and expert consensus (Delphi panel) will be developed for healthcare professionals (i.e., trained psychologists and psychiatrists) and for the certified mindfulness coaches delivering the intervention. Sections to be covered in the SOP manual include: mental health literacy about mood and anxiety disorders; self- and perceived stigma towards mental illness; work–life balance; healthcare system accessibility; early signs of mental disorders; referral to mental health services and intervention options for treatment; mother–child interaction and promotion of secure attachment; social support guidelines; information about biofeedback, including technical details about the wearable devices, health benefits from combining biofeedback with brief group CBT-based psychoeducation interventions; and mindfulness concepts and exercises. With regards to the list of procedures to be used, these include group discussion of vignettes, discussion of illustrative cases (i.e., cases of PPD, poor and adequate mother–child interaction), emotional and cognitive expression exercises, video demonstration, roleplay of mother–child interactions, and demonstration followed by practical exercises on how to use biofeedback devices and interpret biofeedback outputs.

**Figure 1:**
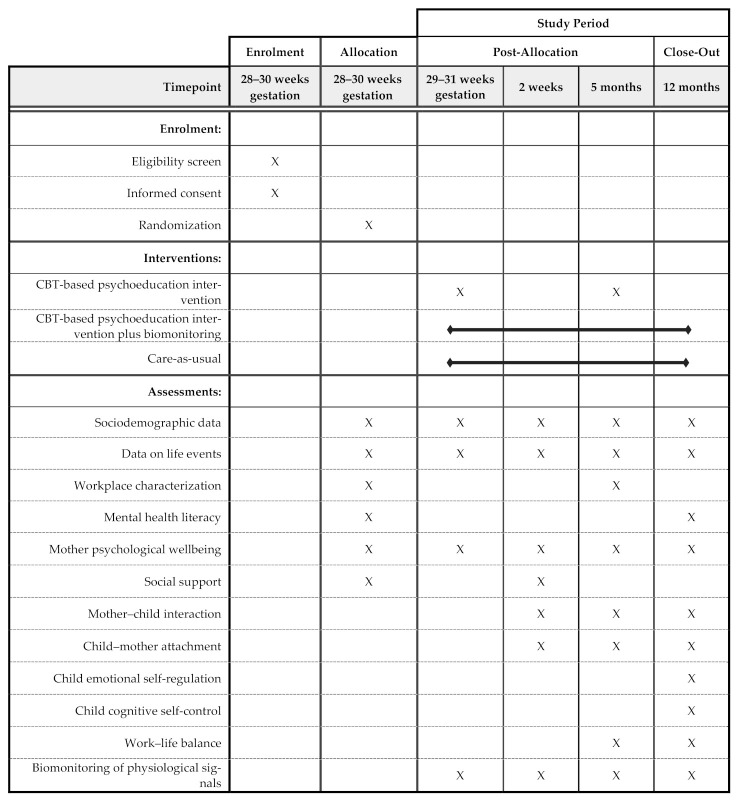
Schedule of enrolment, interventions and assessments of MAMH@WORK project following SPIRIT 2013 checklist.

The RE-AIM (Reach, Effectiveness, Adoption, Implementation, and Maintenance) framework will be used to assess this evidence-based intervention in terms of its potential for translation and public health impact [68]. To assess the reach dimension of this project, the participation rate given as the proportion of invited pregnant women that accepted to participate in the intervention will be calculated. The effectiveness of the intervention will be assessed through a three-arm randomized controlled trial (Figure 1). The intervention group 1 will go through CBT-based psychoeducation sessions (including mindfulness e-training) by formally trained psychiatrists and psychologists; the intervention group 2 will benefit from the same CBT-based psychoeducation sessions (with mindfulness e-training) in addition to a biofeedback intervention; the control group will receive the care-as-usual from their health units. The development of a website for the project is planned and will include public and private areas. The public area will be accessible to the public and will contain information on mental health literacy, a description of the project’s objectives and its progress. Each participant allocated to the intervention arms will be provided with credentials to access her own private area. This area will contain personal, biomonitoring data (if applicable) and mindfulness e-training materials. Progressive Web Apps mimicking the website will be developed for smartphone use (Android and iOS).

Throughout the project, several physical and mental health outcomes will be measured and compared across groups. Since this intervention will be delivered by members of the research team, the adoption dimension (i.e., settings and staff that agree to deliver the intervention) does not apply in this case. However, the adequacy of the syllabus and a set of progress objectives (e.g., number of the psychoeducation intervention hours delivered, costs associated, and number of hours of online mindfulness exercises completed) will be used as a measure of its implementation. Finally, maintenance at both individual and setting levels will be measured at the last moment of data collection respectively by (a) the extent to which attendees maintain behavioral change after participating in the intervention and (b) the willingness of health unit managers to provide their users with this intervention.

Life events with potential confounding effects at the time of effectiveness assessment include becoming unemployed, diagnosis of cancer and life-limiting illnesses, newborn death, complications during delivery that severely impair mother and/or child, gestational diabetes, preeclampsia and eclampsia diagnosis, as well as losing the spouse, either by getting divorced or after death. See Statistical Analyses subsection for further details on how these will be handled.

### 2.4. Data Collection and Variables under Study

Data collection will be performed through self-administered questionnaires, wearable devices for biomonitoring physiological signals (in association with a smartphone app), and direct observations of mother–child interactions. It will be conducted on five different moments (Figure 1): baseline (t_0_; 28–30 gestation weeks), one week after enrolment (t_1_; 29–31 gestation weeks), two weeks postpartum (t_2_), five months postpartum (t_3_), and twelve months postpartum (t_4_). Data collection will be performed by members of the research team. Women in the intervention group with biofeedback will wear a smartwatch to biomonitor several physiological signals combined with the use of a sleep analyzer device (for details, see Table 1). Both devices will communicate through Bluetooth with a datahub and send physiological data to the supplier’s cloud. The server will communicate on a regular basis, via a secure oAuth2 authentication method, with the supplier’s cloud and retrieve all data to be stored in a relational database including wearables-collected information, questionnaires filled in by the participants and health data gathered by healthcare professionals. Health indices computed out of these data will be made available to participants in their website/app private areas, alongside trend graphics and other simple although insightful data visualizations of relevant information such as sleep pattern, accelerometry, pulse rate, pulse rate variability, among others. The system to be developed will also support the project throughout its course, namely by generating reports on its evolution (e.g., successful contacts), listing scheduled contacts, and asking participants to fill in questionnaires, among others.

Strategies to reduce attrition during the experimental period will be implemented. At recruitment, pregnant women will be provided with detailed information about the project, including the objectives and participant’s role in the study. During post-allocation period, referrals to other healthcare professionals will be performed as necessary, and the required logistic support for data collection will be provided, such as scheduling suitable data collection appointments [69]. Reminders will be sent to enrolled women one week before the scheduled date of each data collection moment. Women in the intervention group with biomonitoring of physiological signals will receive tailored health-related information based on data collected through wearable devices (biofeedback), whereas women in both the control and intervention arms will receive general health information via a smartphone app (also accessible in the private area of the project’s website). This app will be developed specifically for MAMH@WORK project and will be available for download by all women enrolled in the study. It will mirror information available at the website and facilitate communication between the research team and participants. 

The main outcomes that will be used to assess the effectiveness of the intervention are women’s psychological wellbeing (mood disorders and anxiety disorders, assessed by the Hospital Anxiety and Depression Scale/HADS and by the Edinburgh Postnatal Depression Scale/EPDS), subjective wellbeing (Self-Report of Happiness), quality of mother–child interactions (assessed by the CARE-Index) and child-mother attachment (assessed by the Strange Situation Procedure and by the Postpartum Bonding Questionnaire/PBQ). Secondary outcomes include child emotional self-regulation and cognitive self-control; women’s job engagement and presenteeism; and women’s physiological signals (pulse rate, pulse rate variability, and sleep patterns).

In order to characterize the sample and adjust outcome indicators, sociodemographic data will be collected and will include women’s age, marital status, household size, educational level, main occupation, monthly income, and child’s gender. Women will be asked to characterize their workplace (e.g., company size, work–life balance policies, and workload) and to provide data on life events, such as previous miscarriages, divorce/loss of spouse, death of a close relative, serious illness diagnosis (of the mother, the child or of a close relative), moving out, and changes in household income. Additional moderation or mediation variables to be collected are mental health literacy, stress-related coping style, resilience, and quality of relationship with partner/husband, perceived support from partner/husband in childcare, kindergarten-related variables (e.g., time per day, and satisfaction with care), and job satisfaction before maternity leave.

All questionnaires to assess the constructs under study were selected taking into account their psychometric properties for the Portuguese population. Table 1 provides an overview of the instruments to be used under MAMH@WORK project.

### 2.5. Statistical Analyses

Statistical analyses will be performed using IBM/SPSS 26.0 (or a more recent version at the time of data analysis) (IBM Corporation, NY, USA) [70] and R Software (R Foundation for Statistical Computing, Vienna, Austria) [71]. For all statistical analyses, the significance level will be set to α = 0.05.

Two strategies for data analysis will be performed: intention-to-treat (ITT) and per-protocol (PP) analyses. ITT considers all participants allocated to arms at the baseline, thus providing a more conservative estimate of the treatment effect, allowing for noncompliance and protocol deviations. In this case, the last observation carried forward method will be used to deal with missing data. PP analysis includes the subset of participants who completed all evaluations. Results from both types of analyses will be reported when comparing control and intervention groups. For effect size estimation, only PP results will be used, since these allow the identification of the treatment effect under optimal conditions, i.e., without major deviations from the protocol. Sensitivity analyses will be carried out to identify potential effects of data strata and missing data on the outcome.

Univariate description of sociodemographic and outcome variables will be performed by providing central tendency (mean and median) and dispersion (standard deviation and range) measures for each moment of data collection. Normality tests (Kolmogorov–Smirnov test or Shapiro–Wilk test) in combination with kurtosis and symmetry analyses will be used to assess the distribution of the variables under study. Levene´s test will be used to assess homoscedasticity. Baseline between-groups comparisons, i.e., control versus intervention groups, will be tested through ANOVA or its non-parametric equivalent (Kruskal–Wallis) for continuous variables, and chi-square test for categorical variables. Mixed model repeated measure (MMRM) analyses will be used for the analyses of main and secondary outcomes, comparing between control and intervention groups and adjusting for relevant covariates (including potential confounding factors). Mediation and moderation analyses will be explored using structural equation modelling. Conceptually and statistically relevant variables will be selected to adjust the models. The size of the intervention effect will be assessed by calculating Cohen’s *d* coefficient [98] Cramer’s V, and odds ratio.

### 2.6. Ethics Approval and Consent to Participate

This study follows the Code of Ethics of the Declaration of Helsinki [99], and it will comply with the applicable national and EU legislation on ethical principles, privacy and data protection. More specifically, it will comply with (a) Directive 2002/58/EC on the processing of personal data and protection of privacy in the electronic communications sector (Directive on privacy and electronic communications) of the European Parliament and of the Council of 12 July 2002, and the (b) EU Regulation 679/2016 on the protection of natural persons with regard to the processing of personal data and on the free movement of such data, and repealing Directive 95/46/EC (General Data Protection Regulation). All researchers involved in the study will sign a confidentiality statement before recruitment or enrolment of pregnant women in the study. A referral plan will be developed to assist researchers in referring women to health services during the project whenever necessary. All participants will be asked to sign a data agreement form and an informed consent form (ICF) before their and of their babies enrolment in the study. The later document will include a detailed explanation of the objectives and methods of the study, as well as their right to refuse or quit their participation at any time. Women who agree to participate in the study also agree with their babies’ participation as stated in the ICF. Delphi panel members will also be asked to read and, in case of agreement, to sign an ICF before their enrolment in the study. Participants will receive the devices for physiological data collection free-of-charge.

The research protocol was approved by the Ethics Committee of the Lisbon Academic Medical Centre of the University of Lisbon. Selection and recruitment of participants will only start after the ethical approval of the Regional Health Administration of Lisbon and Tagus Valley and of the administration board of each healthcare unit. Data confidentiality will be guaranteed and its access will only be granted to team members of the MAMH@WORK project.

## 3. Discussion

The transition to motherhood encompasses numerous changes that require continuous psychosocial adjustment. This places mothers-to-be and recent mothers at an increased risk of developing mental disorders [2,3,4]. At the same time, the perinatal period is a window of opportunity for promoting women’s health/wellbeing and enhancing mother-infant relationships, since these aspects are routinely monitored by healthcare professionals. Here we describe a community-based randomized study protocol to assess the effectiveness of a CBT-based psychoeducation intervention, with or without a biofeedback system, targeting pregnant women at the time of recruitment. MAMH@WORK goes beyond similarly-themed projects (e.g., [100,101]) by adopting an integrated approach in which mother–child relationship, child behavioral, and emotional and cognitive regulation development are addressed, and so is the return to work. Moreover, traditional methods for effectiveness assessment of the intervention are combined with technology-driven approaches providing women with biofeedback on selected physiological indicators. Taking this into consideration, MAMH@WORK emerges as a strategic project for the prevention of mental disorders and for the promotion of mental health and wellbeing during a very challenging period for women and their spouses. Importantly, the intervention will be delivered by trained psychiatrists and psychologists who will follow the referral plan whenever needed, thus contributing for no relevant risks of this intervention to the mother and her child.

Several challenges are anticipated. Recruitment of pregnant women will take place at two different settings (primary health care units and private clinics) and thus, two distinct sociodemographic groups are expected to be enrolled, namely, in terms of their income: pregnant women from high income families have the financial capacity to pay for obstetric appointments at a private clinic, whereas women from low income families will mainly be followed during pregnancy at primary health care centers and public hospitals. Thus, the added-value of this intervention must be stratified according to socioeconomic characteristics. In addition, since the assessment of effectiveness requires repeated measures through time, several strategies to reduce attrition are planned (e.g., periodic telephone contacts, a webpage for project-related communication purposes, and a smartphone app). 

This is a small-scale project to be conducted in the Lisbon Metropolitan Area, and thus it will not mirror the Portuguese reality as a whole. However, if it proves effective in the promotion of maternal mental health/wellbeing, we will scale-up MAMH@WORK to the nation-wide level by establishing a strong network with primary healthcare units and obstetricians from other Portuguese metropolitan areas. This would also contribute to the sustainability of the project beyond its closure, in tandem with several other actions that are planned to ensure that the outcomes can be used after the project ends. For example, expected results will contribute to bring together several stakeholders, including healthcare professionals (i.e., general practitioners, obstetricians, psychiatrists, psychologists, and nurses), employers, and organizational leaders, and it will ultimately inform policy makers and support the elaboration of policy briefs regarding work–life balance and perinatal mental health promotion in the workplace. In addition to policy briefs, a training program on perinatal mental health and organizational leadership styles supporting healthy workplaces will be specifically designed for staff at all levels within the organizations (top-level leaders, intermediate-level leaders, employees). The latter will include a good practices manual for mental health promotion at the workplace. 

## 4. Conclusions

Overall, the expected impacts of MAMH@WORK project will ultimately contribute to meet national and international strategic goals. These include (a) to implement mental health promotion programs and to set early childhood and adolescence mental health as a priority in health agendas, as disclaimed in the current Portuguese National Mental Health Plan [102], (b) to increase health gains in the workplace by monitoring and promoting mental health, as well as to promote healthy workplaces, as defined in the current Portuguese National Occupational Health Plan [103], (c) to promote overall health and wellbeing of women and their children in a sensitive phase of their lives, which is in line with the EU Horizon 2020 societal challenges and the UN Sustainable Development Goal 3 (SDG 3), (d) to empower women and promote their participation in the labour market (SDG 1, SDG 5), with a potential positive impact in fertility rates, and finally, (e) to support smart, sustainable and inclusive economic growth (SDG 8) as outlined in the Europe 2020 Strategy by the European Commission [104].

## Figures and Tables

**Table 1:** Instruments to be used in the effectiveness assessment of the intervention.

Questionnaire/Measure Scale	Description	Timing of Assessment
Mental Health Literacy		
Mental Health Literacy Scale (MHLS) [72]	MHLS is a 35-item questionnaire that assesses six attributes of mental health literacy: recognition of disorders, knowledge on how to seek mental health information, knowledge on risk factors and causes, knowledge of self-treatment, knowledge of professional help available, and attitudes that promote recognition and appropriate help-seeking. High scores indicate high levels of mental health literacy. Instrument validated for the Portuguese population [73].	Pregnancy Postpartum
**Psychological Wellbeing, Resilience and Stress-related Coping Style**		
Hospital Anxiety and Depression Scale (HADS) [74]	HADS is a 14-item screening tool composed of two subscales, one for anxiety and other for depression. High scores indicate the presence of a mood disorder. Instrument validated for the Portuguese population [75].	Pregnancy
Edinburgh Postnatal Depression Scale (EPDS) [76]	EPDS is a 10-item questionnaire that screens for depression in pregnant women and during the first postpartum year. Depressed women score high in EPDS. Instrument validated for the Portuguese population [77].	Postpartum
Kennerley Blues Scale (KBS) [9]	KBS is an 18-item screening tool for postpartum blues. Women are asked to rate current emotions according to how different they feel compared to usual. It is composed of seven clusters: primary blues, reservation, hypersensitivity, depression, despondency, retardation, and decreased self-confidence.	Postpartum
Connor–Davidson Resilience Scale (CD-RISC) [78]	The original CD-RISC is a 25-item scale that measures resilience as the ability of overcoming adversity and stress coping ability. Two shorter versions of this scale are authorized for use, CD-RISC-10 and CD-RISC-2. CD-RISC-25 [79] and CD-RISC-10 [80] have been validated for the Portuguese population. In this study, either the CD-RISC-10 or the CD-RISC-2 (to be validated in this study if applicable) will be used.	Pregnancy Postpartum
Stress-Related Coping Style (BRIEF COPE) [81]	The BRIEF COPE characterizes coping styles and strategies following Lazarus & Folkman’s model [82] and the behavioral self-regulation model.	Pregnancy Postpartum
Self-Report on Happiness [83]	Self-report single-item on happiness to be measured in a daily basis via the smartphone application.	Pregnancy Postpartum
**Social Support and Marital Satisfaction**		
Postpartum Support Questionnaire (PSQ) [84]	PSQ is a 34-item scale composed of four subscales for perceived social support assessment: material support, emotional support, informational support, and comparison support. Respondents are asked to score how important support is perceived to be and how much support is received.	Postpartum
Marital Life Areas Satisfaction Evaluation Scale (EASAVIC) [85]	The EASAVIC (Escala de Avaliação da Satisfação em Áreas da Vida Conjugal) is a 44-item self-administered scale that assesses five dimensions of functional relationship (instrumental components of the relationship) and five dimensions of romantic affects. Developed, tested and validated for the Portuguese population.	Pregnancy Postpartum
**Child-mother Attachment, Child Emotional Self-regulation and Cognitive Self-control**		
The Strange Situation Procedure	This task involves a sequence of eight 3-min episodes designed to place moderate although increasing levels of stress on infants (i.e., unfamiliar play room, interacting with a stranger, and two separations from the mother). Videotapes of infants’ attachment behavior during the Strange Situation Procedure will be scored by trained, reliable coders following the procedures developed by Ainsworth and colleagues [86]. Infants will be classified as either securely attached (B), insecure–avoidant (A), insecure–ambivalent (C), or disorganized–disoriented (D).	Postpartum
Postpartum Bonding Questionnaire (PBQ) [87]	PBQ is a screening tool for mother-child bonding impairment. Women are asked to rate how often they agree with several statements on their feelings or attitudes towards their child. It is composed of four subscales: impaired bonding, rejection and anger, anxiety about care, and risk of abuse. High scores indicate impaired bonding. Instrument validated for the Portuguese population [88].	Postpartum
Questionnaire on the Difference Imaginary Baby vs. Real Baby (QDIBRB) [89]	QDIBRB-30 screens for differences between mother’s prenatal and postnatal perceptions in three dimensions: child positive emotional expressions, mother’s fears about behavioral meaning, and child appealing behavior. Respondents indicate their agreement with each statement in a 5-point Likert scale ranging from completely disagree to completely agree. High scores indicate large differences between imaginary and real baby. Developed, tested and validated for Portuguese women.	Postpartum
Schedule of Growing Skills II (SGS-II) [90]	SGS-II is a screening tool for psychomotor development that considers nine areas: passive posture, active posture, locomotion, manipulation, visual, hearing and language, speech and language, interactive social, self-care social and cognition. Children are classified as having an adequate or delayed development. Instrument validated for the Portuguese population [91].	Postpartum
Toy Removal Task [92]	2-min toy removal episode preceded by 90 s of playing between mother and child. During removal and after returning the toy, mothers will be instructed to stay non-interactive and assume a neutral expression.	Postpartum
Object Permanence	Object permanence is one of the cognitive developmental milestones. This experiment involves hiding a toy from the child to assess if the child would look for it (ca. 8 months old) or retrieving the toy at location B even though it was previously hidden several times at location A (A-not-B experiment; ca. 12 months old).	Postpartum
Child–Adult Relationship Experimental Index (CARE-Index) [93]	The CARE-Index assesses mother–infant interaction from birth to about two years of age. Mothers and infants are videotaped for 5 min during an unstructured free play session. A standard set of age-appropriate toys are arranged on a quilt on the floor of a laboratory playroom and mothers are instructed to play with their infant as they usually do. The CARE-Index system focuses on seven aspects of the infant´s and mother’s interactive behavior: facial expression, verbal expression, position and body contact, affection, turn-taking contingencies, control, and choice of activity.	Postpartum
**Work** **–** **life Balance**		
The Utrecht Work Engagement Scale (UWES) [94]	UWES-17 is a three-dimensional scale that assesses vigor, dedication and absorption. Respondents are asked to score several emotions on a 7-point frequency scale. High scores indicate high engagement levels. Instrument validated for the Portuguese population [95].	Postpartum
Work–Family Balance Scale (WFB) [96]	WFB is a 5-item scale that assesses the extent to which women were able to balance work and their family life. High scores indicate good ability to balance work and family life demands.	Postpartum
Presenteeism questions of the World Health Organization’s Heath and Work Performance Questionnaire (HPQ) [97]	These questions are part of the full HPQ and measure the actual performance versus possible performance. High scores indicate low amount of lost performance, i.e., low presenteeism.	Postpartum
Self-reported absenteeism	Self-reported absenteeism is measured by asking individuals if they have missed work in the last year, how many days and what was the reason for that.	Postpartum
**Biomonitoring of Physiological Signals**		
Pulse rate (PR) Pulse rate variability (PRV) Body motion generated by the ejection of the blood at each cardiac cycle (Ballistocardiography, BCG) Peripheral capillary oxygen saturation (SpO_2_) Sleep patterns (SP) Physical activity (PA) tracking and sleep-time movement tracking Electrocardiogram (ECG)	PR, PRV SP, BCG, and PA will be monitored using a Hybrid Smartwatch. The Hybrid Smartwatch combined with the Sleep Analyzer will allow sleep-stage characterization. A sleep score based on six factors is also calculated: sleep duration, sleep regularity, time to sleep, sleep depth, frequency of sleep interruptions, and time to get up. Women will be invited to ask for an ECG once a week using the Hybrid Smartwatch.	Pregnancy Postpartum

## References

[1] Committee on Obstetric Practice (2015). The American College of Obstetricians and Gynecologists Committee Opinion No. 630. Screening for perinatal depression. Obstet. Gynecol..

[2] Salmela-Aro K., Nurmi J.E., Saisto T., Halmesmäki E. (2001). Goal reconstruction and depressive symptoms during the transition to motherhood: Evidence from two cross-lagged longitudinal studies. J. Pers. Soc. Psychol..

[3] Miller L.J. (2002). Postpartum depression. JAMA.

[4] Seyfried L.S., Marcus S.M. (2003). Postpartum mood disorders. Int. Rev. Psychiatry.

[5] Skouteris H., Cash T.F. (2012). Pregnancy: Physical and Body Image Changes. Encyclopedia of Body Image and Human Appearance.

[6] Watson B., Fuller-Tyszkiewicz M., Broadbent J., Skouteris H. (2015). The meaning of body image experiences during the perinatal period: A systematic review of the qualitative literature. Body Image.

[7] Brazelton T.B., Cranmer B.G. (1990). The Earliest Relationship.

[8] Stern D.N. (1991). Maternal representations: A clinical and subjective phenomenological view. Infant Ment. Health J..

[9] Kennerley H., Gath D. (1989). Maternity blues. I. Detection and measurement by questionnaire. Br. J. Psychiatry.

[10] Watanabe M., Wada K., Sakata Y., Aratake Y., Kato N., Ohta H., Tanaka K. (2008). Maternity blues as predictor of postpartum depression: A prospective cohort study among Japanese women. J. Psychosom. Obstet. Gynecol..

[11] Reck C., Stehle E., Reinig K., Mundt C. (2009). Maternity blues as a predictor of DSM-IV depression and anxiety disorders in the first three months postpartum. J. Affect. Disord..

[12] Wisner K.L., Chambers C., Sit D.K.Y. (2006). Postpartum depression—A major public health problem. JAMA.

[13] O’Hara M.W., McCabe J.E. (2013). Postpartum depression: Current status and future directions. Annu. Rev. Clin. Psychol..

[14] American Psychiatric Association (2013). Diagnostic and Statistical Manual of Mental Disorders: DSM-5.

[15] Breedlove G., Fryzelka D. (2011). Depression screening during pregnancy. J. Midwifery Womens Health.

[16] Fuller-Tyszkiewicz M., Skouteris H., Watson B.E., Hill B. (2013). Body dissatisfaction during pregnancy: A systematic review of cross-sectional and prospective correlates. J. Health Psychol..

[17] Paulson J.F., Bazemore S.D. (2010). Prenatal and postpartum depression in fathers and its association with maternal depression. JAMA.

[18] Letourneau N.L., Dennis C.-L., Benzies K., Duffett-Leger L., Stewart M., Tryphonopoulos P.D., Este D., Watson W. (2012). Postpartum depression is a family affair: Addressing the impact on mothers, fathers, and children. Issues Ment. Health Nurs..

[19] Burke L. (2003). The impact of maternal depression on familial relationships. Int. Rev. Psychiatry.

[20] Feldman R., Weller A., Zagoory-Sharon O., Levine A. (2007). Evidence for a neuroendocrinological foundation of human affiliation: Plasma oxytocin levels across pregnancy and the postpartum period predict mother-infant bonding. Psychol. Sci..

[21] Galbally M., Lewis A.J., van Ijzendoorn M., Permezel M. (2011). The role of oxytocin in mother-infant relations: A systematic review of human studies. Harv. Rev. Psychiatry.

[22] Kumar R.C. (1997). Anybody’s child: Severe disorders of mother-to- infant bonding. Br. J. Psychiatry.

[23] Taylor A., Atkins R., Kumar R., Adams D., Glover V. (2005). A new Mother-to-Infant Bonding Scale: Links with early maternal mood. Arch. Womens Ment. Health.

[24] Yoshida K., Yamashita H., Conroy S., Marks M., Kumar C. (2012). A Japanese version of Mother-to-Infant Bonding Scale: Factor structure, longitudinal changes and links with maternal mood during the early postnatal period in Japanese mothers. Arch. Womens Ment. Health.

[25] Hornstein C., Trautmann-Villalba P., Hohm E., Rave E., Wortmann-Fleischer S., Schwarz M. (2006). Maternal bond and mother-child interaction in severe postpartum psychiatric disorders: Is there a link?. Arch. Womens Ment. Health.

[26] O’Higgins M., Roberts I.S.J., Glover V., Taylor A. (2013). Mother-child bonding at 1 year; Associations with symptoms of postnatal depression and bonding in the first few weeks. Arch. Womens Ment. Health.

[27] Righetti-Veltema M., Conne-Perréard E., Bousquet A., Manzano J. (2002). Postpartum depression and mother-infant relationship at 3 months old. J. Affect. Disord..

[28] Benoit D. (2004). Infant-parent attachment: Definition, types, antecedents, measurement and outcome. Paediatr. Child Health.

[29] Dias C.C., Figueiredo B. (2015). Breastfeeding and depression: A systematic review of the literature. J. Affect. Disord..

[30] Gress-Smith J.L., Luecken L.J., Lemery-Chalfant K., Howe R. (2012). Postpartum depression prevalence and impact on infant health, weight, and sleep in low-income and ethnic minority women and infants. Matern. Child Health J..

[31] Netsi E., Pearson R.M., Murray L., Cooper P., Craske M.G., Stein A. (2018). Association of persistent and severe postnatal depression with child outcomes. JAMA Psychiatry.

[32] Brennan P.A., Hammen C., Andersen M.J., Bor W., Najman J.M., Williams G.M. (2000). Chronicity, severity, and timing of maternal depressive symptoms: Relationships with child outcomes at age 5. Dev. Psychol..

[33] Stein A., Pearson R.M., Goodman S.H., Rapa E., Rahman A., McCallum M., Howard L.M., Pariante C.M. (2014). Effects of perinatal mental disorders on the fetus and child. Lancet.

[34] Sciberras E., Ukoumunne O.C., Efron D. (2011). Predictors of parent-reported attention-deficit / hyperactivity disorder in children aged 6–7 years: A national longitudinal study. J. Abnorm. Child Psychol..

[35] Park S., Cho S.-C., Kim J.-W., Shin M.-S., Yoo H.-J., Min S., Hyun D.H., Cheong J.H., Kim B.-N. (2014). Differential perinatal risk factors in children with attention-deficit/ hyperactivity disorder by subtype. Psychiatry Res..

[36] Kingston D., Kehler H., Austin M., Mughal M.K., Wajid A., Vermeyden L., Benzies K., Brown S., Stuart S., Giallo R. (2018). Trajectories of maternal depressive symptoms during pregnancy and the first 12 months postpartum and child externalizing and internalizing behavior at three years. PLoS ONE.

[37] Pearson R.M., Bornstein M.H., Cordero M., Scerif G., Mahedy L., Evans J., Abioye A., Stein A. (2016). Maternal perinatal mental health and offspring academic achievement at age 16: The mediating role of childhood executive function. J. Child Psychol. Psychiatry.

[38] Letourneau N.L., Tramonte L., Willms J.D. (2013). Maternal depression, family functioning and children’s longitudinal development. J. Pediatr. Nurs..

[39] Dennis C.L., Dowswell T. (2013). Psychosocial and psychological interventions for preventing postpartum depression (Review). Cochrane Database Syst. Rev..

[40] European Network for Workplace Health Promotion (2018). Luxembourg Declaration on Workplace Health Promotion in the European Union.

[41] Popli S., Rizvi I.A. (2016). Drivers of employee engagement: The role of leadership style. Glob. Bus. Rev..

[42] Walton M., Kinder A., Hughes R., Cooper C.L. (2008). In consideration of a toxic workplace: A suitable place for treatment. Employee Well-Being Support a Workplace Resource.

[43] Jurviste U., Prpic M., Sabbati G. (2019). Maternity and Paternity Leave in the EU.

[44] Jahoda M. (1982). Employment and Unemployment: A Social-Psychology Analysis.

[45] Nichols M.R., Roux G.M. (2004). Maternal perspectives on postpartum return to the workplace. J. Obstet. Gynecol. Neonatal Nurs..

[46] Greenhaus J.H., Beutell N.J. (1989). Sources of conflict between work and family roles. Acad. Manag. Rev..

[47] PORDATA Idade Média da Mãe ao Nascimento do Primeiro Filho. https://www.pordata.pt/Portugal/Idade+média+da+mãe+ao+nascimento+do+primeiro+filho-805.

[48] Gilbreath B., Benson P.G. (2004). The contribution of supervisor behaviour to employee psychological well-being. Work Stress.

[49] Skakon J., Nielsen K., Borg V., Guzman J. (2010). Are leaders’ well-being, behaviours and style associated with the affective well-being of their employees? A systematic review of three decades of research. Work Stress.

[50] European Union (2019). Directive of the European Parliament and of the Council on Work-Life Balance for Parents and Carers and Repealing.

[51] Werner E., Miller M., Osborne L.M., Kuzava S., Monk C. (2015). Preventing postpartum depression: Review and recommendations. Arch. Womens Ment. Health.

[52] O’Connor E., Senger C.A., Henninger M.L., Coppola E., Gaynes B.N. (2019). Interventions to prevent perinatal depression—Evidence report and systematic review for the US Preventive Services Task Force. JAMA.

[53] Sangsawang B., Wacharasin C., Sangsawang N. (2019). Interventions for the prevention of postpartum depression in adolescent mothers: A systematic review. Arch. Womens Ment. Health.

[54] De Witte N.A.J., Buyck I., Van Daele T. (2019). Combining biofeedback with stress management interventions: A systematic review of physiological and psychological effects. Appl. Psychophysiol. Biofeedback.

[55] Association for Applied Psychophysiology and Biofeedback About Biofeedback—AAPB. https://www.aapb.org/i4a/pages/index.cfm?pageid=3463.

[56] Beckham A.J., Greene T.B., Meltzer-Brody S. (2013). A pilot study of heart rate variability biofeedback therapy in the treatment of perinatal depression on a specialized perinatal psychiatry inpatient unit. Arch. Womens Ment. Health.

[57] Kudo N., Shinohara H., Kodama H. (2014). Heart rate variability biofeedback intervention for reduction of psychological stress during the early postpartum period. Appl. Psychophysiol. Biofeedback.

[58] Dhillon A., Sparkes E., Duarte R.V. (2017). Mindfulness-based interventions during pregnancy: A systematic review and meta-analysis. Mindfulness.

[59] Shi Z., MacBeth A. (2017). The effectiveness of mindfulness-based interventions on maternal perinatal mental health outcomes: A systematic review. Mindfulness.

[60] Kabat-Zinn J. (2003). Mindfulness-based interventions in context: Past, present, and future. Clin. Psychol. Sci. Pract..

[61] Howarth A., Smith J.G., Perkins-Porras L., Ussher M. (2019). Effects of brief mindfulness-based interventions on health-related outcomes: A systematic review. Mindfulness.

[62] Hofmann S.G., Sawyer A.T., Witt A.A., Oh D. (2010). The effect of mindfulness-based therapy on anxiety and depression: A meta-analytic review. J. Consult. Clin. Psychol..

[63] Pan W.-L., Chang C.-W., Chen S.-M., Gau M.-L. (2019). Assessing the effectiveness of mindfulness-based programs on mental health during pregnancy and early motherhood—A randomized control trial. BMC Pregnancy Childbirth.

[64] Strazdins L., Shipley M., Broom D.H. (2007). What does family-friendly really mean? Wellbeing, time, and the quality of parents’ jobs. Aust. Bull. Labour.

[65] Leigh B., Milgrom J. (2008). Risk factors for antenatal depression, postnatal depression and parenting stress. BMC Psychiatry.

[66] O’Hara M.W., Swain A.M. (1996). Rates and risk of postpartum depression—A meta-analysis. Int. Rev. Psychiatry.

[67] Pocock S.J., Simon R. (1975). Sequential treatment assignment with balancing for prognostic factors in the controlled clinical trial. Biometrics.

[68] Glasgow R.E., Klesges L.M., Dzewaltowski D.A., Estabrooks P.A., Vogt T.M. (2006). Evaluating the impact of health promotion programs: Using the RE-AIM framework to form summary measures for decision making involving complex issues. Health Educ. Res..

[69] Robiner W.N. (2005). Enhancing adherence in clinical research. Contemp. Clin. Trials.

[70] IBM Corporation (2019). IBM SPSS Statistics for Windows, Version 26.0.

[71] R Core Development Team (2014). R: A Language and Environment for Statistical Computing.

[72] O’Connor M., Casey L. (2015). The Mental Health Literacy Scale (MHLS): A new scale-based measure of mental health literacy. Psychiatry Res..

[73] Rocha I. (2016). Adaptação e Validação da Escala Mental Health Literacy para a População Portuguesa [Adaptation and Validation of the Mental Health Literacy Scale for the Portuguese Population].

[74] Zigmond A., Snalth R. (1983). The Hospital Anxiety and Depression Scale. Acta Psychiatr. Scand..

[75] Pais-Ribeiro J., Silva I., Ferreira T., Martins A., Meneses R., Baltar M. (2007). Validation study of a Portuguese version of the Hospital Anxiety and Depression Scale. Psychol. Health Med..

[76] Cox J.L., Holden J.M., Sagovsky R. (1987). Detection of postnatal depression: Development of the 10-item Edinburgh Postnatal Depression Scale. Br. J. Psychiatry.

[77] Areias M.E.G., Kumar R., Barros H., Figueiredo E. (1996). Comparative incidence of depression in women and men, during pregnancy and after childbirth: Validation of the Edinburgh Postnatal Depression Scale in Portuguese mothers. Br. J. Psychiatry.

[78] Connor K.M., Davidson J.R.T. (2003). Development of a new resilience scale: The Connor-Davidson Resilience scale (CD-RISC). Depress. Anxiety.

[79] Anjos J.F., Heitor Dos Santos M., Ribeiro M.T., Moreira S. (2019). Connor-Davidson Resilience Scale: Validation study in a Portuguese sample. BMJ Open.

[80] Heitor M.J. (2019). Promoção da Saúde Mental no Trabalho: Estudo Observacional de Determinantes Biopsicossociais e Presentismo.

[81] Carver C.S. (1997). You want to measure coping but your protocol’s too long: Consider the brief COPE. Int. J. Behav. Med..

[82] Lazarus R.S., Folkman S. (1984). Stress, Appraisal, and Coping..

[83] Veenhoven R. Measures of Happiness, World Database of Happiness. http://worlddatabaseofhappiness.eur.nl/hap_quer/hqi_fp.htm.

[84] Logsdon M.C., Usui W., Birkimer J.C., McBride A.B. (1996). The Postpartum Support Questionnaire: Reliability and validity. J. Nurs. Meas..

[85] Narciso I., Costa M.E. (1996). Amores satisfeitos, mas não perfeitos. Cad. Consult. Psicológica.

[86] Ainsworth M.D.S., Blehar M.C., Waters E., Wall S. (1978). Patterns of Attachment: A Psychological Study of the Strange Situation.

[87] Brockington I.F., Fraser C., Wilson D. (2006). The Postpartum Bonding Questionnaire: A validation. Arch. Womens Ment. Health.

[88] Nazaré B., Fonseca A., Canavarro M.C. (2012). Avaliação da ligação parental ao bebé após o nascimento: Análise fatorial confirmatória da versão portuguesa do Postpartum Bonding Questionnaire (PBQ). Laboratório Psicol..

[89] Chagas C.S., Maltez P.M.L., Miranda S.I.S., Justo J.M.R.M. (2015). The “Questionnaire on the Difference Imaginary Baby Vs. Real Baby”: A new instrument for the evaluation of differences between perinatal and postnatal maternal perceptions after delivery. Int. J. Dev. Educ. Psychol..

[90] Bellman M., Lingam S., Aukett A. (2012). Schedule of Growing Skills II: Escala de Avaliação das Competências no Desenvolvimento Infantil II—Dos 0 aos 5 Anos—Manual do Utilizador.

[91] Varajidás C.A., Machado M., Mota M.P., Martins R., Lisboa M.C., Soares I., Sousa S., Leitão J.C. (2017). Psychometric properties of the Schedule of Growing Skills II: Portuguese version. Psychologica.

[92] Stifter C.A., Braungart J.M. (1995). The Regulation of negative reactivity in infancy: Function and development. Dev. Psychol..

[93] Crittenden P.M. (2003). CARE-Index Manual.

[94] Schaufeli W., Bakker A. (2003). UWES—Utrecht Work Engagement Scale.

[95] Teles H., Ramalho N., Ramalho V., Ribeiro S. (2017). Adaptação e Validação da Utrecht Work Engagement Scale (UWES) aplicada a Assistentes Sociais em Portugal. Rev. Port. Investig. Comport. Soc..

[96] Hill E.J., Hawkins A.J., Ferris M., Weitzman M. (2001). Finding an extra day a week: The positive influence of perceived job flexibility on work and family life balance. Fam. Relat..

[97] Kessler R.C., Barber C., Beck A.L., Berglund P.A., Cleary P.D., McKenas D., Pronk N.P., Simon G.E., Stang P.E., Üstün T.B. (2003). The World Health Organization Health and Work Performance Questionnaire (HPQ). J. Occup. Environ. Med..

[98] Cohen J. (1977). Statistical Power Analysis for the Behavioral Sciences.

[99] World Medical Association (2013). World Medical Association Declaration of Helsinki: Ethical principles for medical research involving human subjects. JAMA.

[100] Fonseca A., Pereira M., Araújo-Pedrosa A., Gorayeb R., Ramos M.M., Canavarro M.C. (2018). Be a Mom: Formative evaluation of a web-based psychological intervention to prevent postpartum depression. Cogn. Behav. Pract..

[101] Marvin R., Cooper G., Hoffman K., Powell B. (2002). The Circle of Security project: Attachment-based intervention with caregiver-pre-school child dyads. Attach. Hum. Dev..

[102] Comissão Técnica de Acompanhamento da Reforma da Saúde Mental (2017). Relatório da Avaliação do Plano Nacional de Saúde Mental 2007–2016 e Propostas Prioritárias para a Extensão a 2020.

[103] Nogueira J.R., Moreira S. (2018). Programa Nacional de Saúde Ocupacional (PNSOC)—Extensão 2018/2020.

[104] European Commission (2010). Europe 2020—A strategy for Smart, Sustainable and Inclusive Growth.

